# Celiac disease and attention-deficit/hyperactivity disorder: a bidirectional Mendelian randomization analysis

**DOI:** 10.3389/fpsyt.2024.1291096

**Published:** 2024-05-29

**Authors:** Jing Chen, Qiaozhen Zhu, Lan Li, Zheng Xue

**Affiliations:** ^1^ Department of Pediatrics, Shanghai Municipal Hospital of Traditional Chinese Medicine, Shanghai University of Traditional Chinese Medicine, Shanghai, China; ^2^ Infection and Immunity Institute and Translational Medical Center, Huaihe Hospital of Henan University, Kaifeng, China; ^3^ Department of Pediatrics, The first affiliated hospital of Zhejiang Chinese Medical University, Hangzhou, China

**Keywords:** celiac disease, attention-deficit/hyperactivity disorder, Mendelian randomization, causality, genetic

## Abstract

**Background:**

Recent observational research suggests a potential link between celiac disease (CeD) and an increased incidence of attention-deficit/hyperactivity disorder (ADHD). However, the genetic relationship between CeD and ADHD remains unclear. In order to assess the potential genetic causality between these two conditions, we conducted a Mendelian randomization (MR) analysis.

**Methods:**

We performed a bidirectional MR analysis to investigate the relationship between CeD and ADHD. We carefully selected single nucleotide polymorphisms (SNPs) from publicly available large-scale genome-wide association studies (GWAS) databases, employing rigorous quality screening criteria. MR estimates were obtained using four different methods: fixed-effect inverse variance weighted (fe-IVW), random-effect inverse variance weighting (re-IVW), weighted median (WM), and MR-Egger. The robustness and reliability of our findings were confirmed through sensitivity analyses, assessment of instrumental variable (IV) strength (F-statistic), and statistical power calculations.

**Results:**

Our MR analyses did not reveal any significant genetic associations between CeD and ADHD (fe-IVW: OR = 1.003, 95% CI = 0.932–1.079, P = 0.934). Similarly, in the reverse direction analysis, we found no evidence supporting a genetic relationship between ADHD and CeD (fe-IVW: OR = 0.850, 95% CI = 0.591–1.221, P = 0.378). Various MR approaches consistently yielded similar results. Sensitivity analysis indicated the absence of significant horizontal pleiotropy or heterogeneity. However, it’s important to note that the limited statistical power of our study may have constrained the causal analysis of the exposure’s influence on the outcome.

**Conclusions:**

Our findings do not provide compelling evidence for a genetic association between CeD and ADHD within the European population. While the statistical power of our study was limited, future MR research could benefit from larger-scale datasets or datasets involving similar traits. To validate our results in real-world scenarios, further mechanistic studies, large-sample investigations, multicenter collaborations, and longitudinal studies are warranted.

## Introduction

Celiac disease (CeD) is an immunological disorder triggered by the consumption of gluten in genetically susceptible individuals (1). Reports suggest that CeD affects approximately 1% of individuals in most populations; however, a significant number of patients remain undiagnosed (1, 2). CeD is a lifetime illness that can manifest at any age (3). It represents a systemic ailment characterized by a complex pathophysiology and diverse clinical presentations. These include not only common nonspecific symptoms like bloating, vomiting, and abdominal pain but also typical gastrointestinal manifestations such as chronic diarrhea and weight loss. Moreover, a wide array of symptoms affecting multiple organs constitute its extra-intestinal presentations (4). Notably, many foods contain the gluten that causes celiac disease (5), and some commonly used food additives (like monosodium glutamate) are also derived from gluten (6, 7). As a result, food contamination with gluten is a common issue, making the issues related to celiac disease all the more critical to consider.

Untreated CeD patients exhibit a prevalence of psychiatric disorders of up to 21% (4). However, the precise mechanisms underlying the pathophysiology and development of behavioral and mental disorders associated with CeD remain a mystery. One hypothesis suggests that the ingestion of gluten and its subsequent breakdown into immunogenic peptides may lead to the leakage of these peptides through the intestinal wall. Subsequently, these peptides could breach the blood-brain barrier, potentially triggering mild brain inflammation (4). These immune pathways may play a role in influencing the development and manifestation of ADHD (8–10).

ADHD, a neurological condition characterized by age-inappropriate levels of inattention and hyperactivity/impulsivity, coupled with functional impairments across various settings, often endures from childhood into adolescence and adulthood (11). Globally, the prevalence of ADHD in individuals under the age of 18 is approximately 5% (12), and over the past three decades, this prevalence has remained stable (13). In recent years, the relationship between ADHD and CeD has garnered significant attention. Several case-control studies have shown no significant difference between individuals with celiac disease (CeD) and control groups concerning the prevalence of ADHD or ADHD-like symptoms (14, 15). Conversely, contrasting findings have been reported in other studies, indicating a notably higher incidence of ADHD among individuals with CeD compared to control groups (16–18). Consequently, the correlation between CeD and ADHD warrants further comprehensive investigation.

Mendelian randomization (MR) is a robust statistical method that employs genetic variables as instrumental variables to discern the genetic relationship between an exposure and an outcome (19). Given the stability of genes post-fertilization, MR analysis is particularly effective in mitigating reverse causality issues (20). Additionally, MR analysis can help mitigate confounding effects by randomly allocating alleles (21). In our study, we investigated the potential association between CeD and ADHD utilizing publicly available large-scale genome-wide association studies (GWAS) datasets through a two-sample bidirectional MR analysis.

## Materials and methods

### Research design and data source

**Figure 1:**
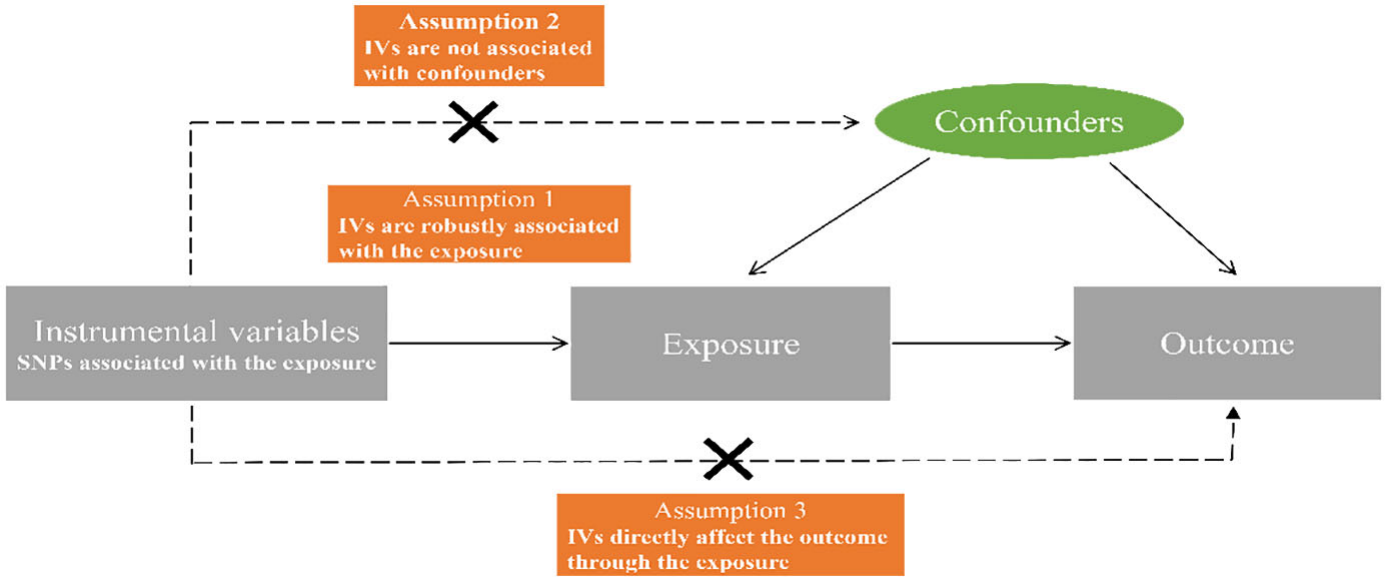
Diagrammatic representation of the Mendelian randomization analysis’s underlying assumptions. SNPs, single nucleotide polymorphisms; IVs, instrumental variables.

In MR research, three crucial assumptions (22) must be satisfied by the instrumental variables (IVs), as illustrated in **Figure 1**: (1) IVs should exhibit a significant correlation with the exposure; (2) IVs should remain unconfounded in the relationship between exposure and outcome; (3) IVs should not exert any influence on the outcome through mechanisms other than the exposure itself.

Our study placed a high priority on using the most recent Genome-Wide Association Study (GWAS) data from published studies or readily accessible GWAS statistics. For CeD, we sourced summary statistics from the MR-base platform (23), a resource developed by Trynka et al. (24) (https://gwas.mrcieu.ac.uk/datasets/ieu-a-1058/). Regarding Attention Deficit/Hyperactivity Disorder (ADHD), we obtained GWAS data from a meta-analysis published within the Psychiatric Genomics Consortium’s dataset (https://pgc.unc.edu/) (25). However, in our attempts to conduct a reverse Mendelian randomization (MR) analysis to explore the relationship between ADHD (exposure) and CeD (outcome), our instrumental variables (IVs) failed to extract relevant information for the outcome variable. Consequently, we switched to another GWAS database for celiac disease, provided by Dubois et al. (26) (https://gwas.mrcieu.ac.uk/datasets/ieu-a-276/). Detailed data can be found in **Supplementary Table 1**.

### Selecting instrument variables

**Figure 2:**
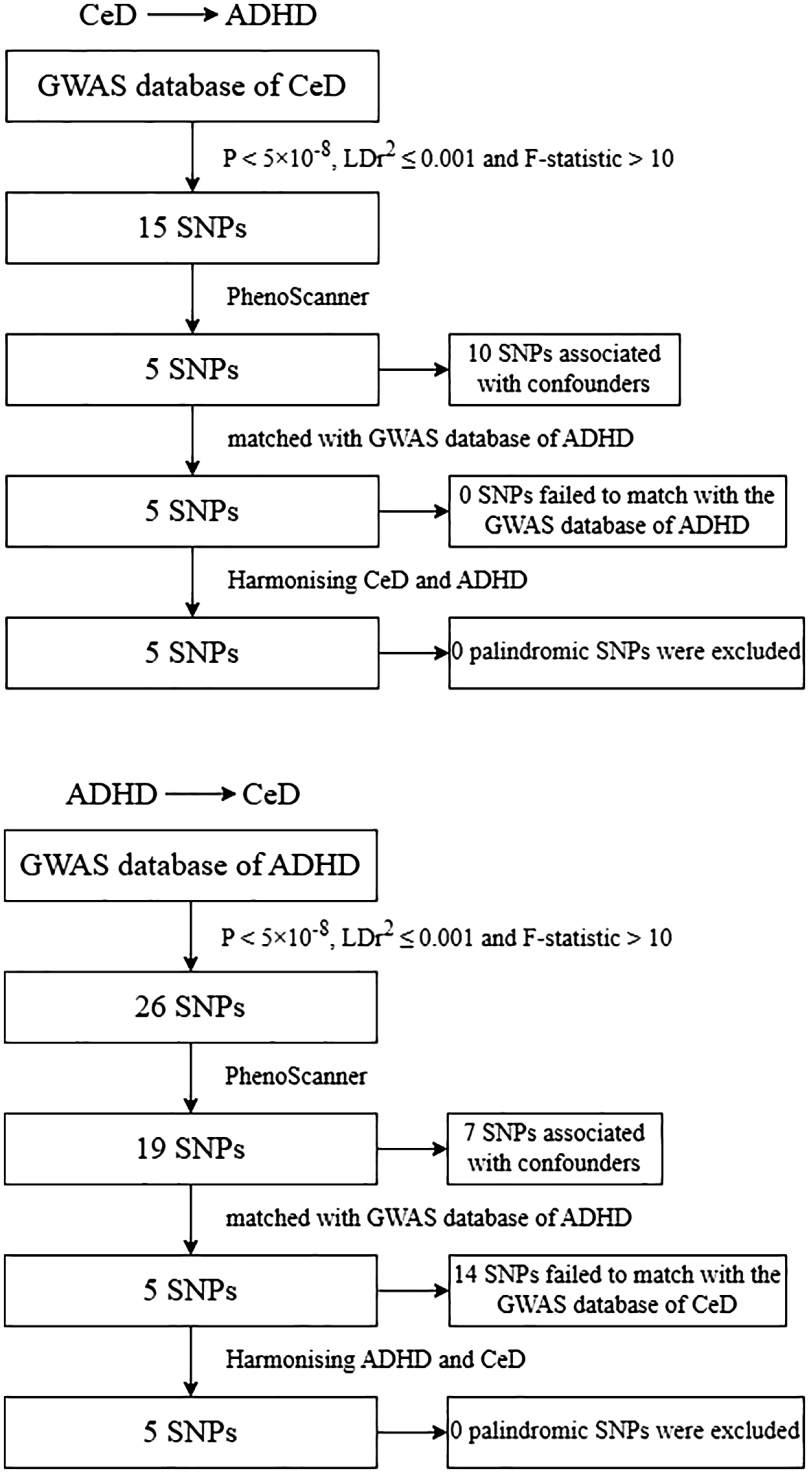
Flowchart of the genetic instruments’ generating and analysis. CeD, celiac disease; ADHD, attention-deficit/hyperactivity; SNP, single nucleotide polymorphism. GWAS, genome‐wide association studies.

The genetic instrument’s production and analysis process is illustrated in **Figure 2**. Initially, we employed the genome-wide significance criterion (P<5 ×10^− 8^) to identify SNPs and subsequently filtered out highly correlated variants with $r^{2}>\;0.001$ to mitigate linkage disequilibrium (LD) within a 10,000KB range (27, 28). For each IV, we assessed its strength using the F-statistic of SNPs, calculated as follows: $F=R^{2}\frac{N-2}{1-R^{2}}$ , where $R^{2}$ represents the proportion of variance in the exposure explained by the genetic instrument, and N signifies the sample size (29). A recommended F-statistic threshold of greater than 10 was utilized to ensure the use of robust genetic instruments (29). R^2^ for the SNP instrument was determined using the formula: $2\times EAF\times \left(1-EAF\right)\times {\text{beta}}^{2}$ , with EAF representing the effect allele frequency and beta indicating the estimated genetic effect on exposure (30). We employed PhenoScanner V2 (www.phenoscanner.medschl.cam.ac.uk) (31) and previous MR studies (32–35) to exclude SNPs associated with potential confounding factors. In the final stage, certain SNPs were excluded either due to their lack of correspondence with data in the GWAS outcome database or the presence of palindromic structures.

### Statistical analysis

We employed four distinct methods to assess the Mendelian Randomization (MR) estimates for the relationship between CeD and ADHD: fixed effect inverse variance weighting (fe-IVW), random effect inverse variance weighting (re-IVW), weighted median (WM), and MR-Egger. The primary statistical model used for aggregating SNP-specific Wald-ratio estimates was fe-IVW (36). This traditional MR method yields robust results when all instrumental variables (IVs) are valid and free from pleiotropic effects (37). In cases with substantial heterogeneity among SNPs, the IVW in the random effects model can provide more dependable estimates (38). The presence of pleiotropy in MR studies can introduce bias and render MR estimates unstable (39). Consequently, as supplementary MR estimations, we applied MR-Egger and WM. The MR-Egger analysis includes an intercept test to detect horizontal pleiotropy (40). Even in the presence of pleiotropic IVs, the MR-Egger technique offers a cautious assessment of causal effects, with resulting statistics resistant to exaggeration (41). The WM method accommodates the possibility of up to 50% of the variables in the SNPs being non-valid instrumental variables (42), allowing for a consistent evaluation of causal effects.

We conducted sensitivity analyses to ensure the reliability of our MR results. In our study, we employed Cochran’s Q statistic (P< 0.05) to identify significant heterogeneity among the estimates of each included SNP (43). Indications of horizontal pleiotropy were evaluated through the MR-Egger regression’s intercept (P< 0.05, suggesting potential horizontal pleiotropy). Likewise, MR-Pleiotropy Residual Sum and Outlier methods (MR-PRESSO) were employed to find outliers and possible horizontal pleiotropy (global test P< 0.05 implies the presence of horizontal pleiotropy) (44). If outliers were detected, they were excluded to obtain a more accurate corrected estimate (44). In addition, the stability of MR estimates after the exclusion of the particular SNP was assessed using the leave-one-out method (45).

In an effort to rule out the possibility of reverse causality, we also changed the outcome and exposure and reran the MR and sensitivity analyses. The R packages “Two Sample MR” and “MR-PRESSO” were used for MR analysis, and R version 4.2.1 was utilized for all studies. Using the online calculator (https://cnsgenomics.shinyapps.io/mRnd/) (46), power calculations were done using the outcome sample size, proportion of cases, R^2^ sum, and a type I error rate of 0.05.

## Result

### Effects of CeD on ADHD

Following a rigorous process for identifying suitable instrumental variables (IVs), we identified 5 SNPs strongly associated with CeD to serve as IVs in our Mendelian Randomization (MR) analysis. These selected SNPs collectively account for 3.24 percent of the variance in CeD across the population. Their F-statistics, presented in **Supplementary Table 2**, ranged from 123.78 to 233.67. The MR results, as detailed in **Supplementary Table 3**, indicate that based on the fe-IVW analysis, no statistically significant causal relationship between CeD and the risk of ADHD was observed (OR = 1.003, 95% CI = 0.932–1.079, P = 0.934). Similarly, the re-IVW (OR = 1.003, 95% CI = 0.909–1.107, P = 0.951), MR-Egger (OR = 1.337, 95% CI = 0.719–2.486, P = 0.426), and WM method (OR = 1.001, 95% CI = 0.903–1.109, P = 0.985) yielded consistent results. Furthermore, Cochran’s Q statistic did not reveal significant heterogeneity in estimating the included SNPs (P = 0.129), and leave-one-out analysis confirmed the stability of our MR estimations (**Supplementary Figure 1B**). The MR-Egger intercept of -0.038 (P = 0.425), as shown in **Supplementary Table 3**, indicates no apparent horizontal pleiotropy. Using MR-PRESSO, no significant horizontal pleiotropy was detected (global test P = 0.179), and our sample exhibited no outliers (**Supplementary Table 3**). However, it is important to note that our study had limited statistical power, with only 5.00% power to detect an association between ADHD and the IVs of CeD (**Supplementary Table 4**).

### Effects of ADHD on CeD

In the reverse MR analysis, 5 SNPs were utilized as IVs for ADHD and collectively accounted for 1.04% of the phenotypic variation. The F-statistics ranged from 331.86 to 810.67, all of which exceeded 10, indicating the absence of weak instrument bias (**Supplementary Table 2**). The results presented in **Supplementary Table 3** revealed no significant causal relationship between ADHD and CeD when employing various methods, including fe-IVW (OR = 0.850, 95% CI = 0.591–1.221, P = 0.378), re-IVW (OR = 0.850, 95% CI = 0.489–1.476, P = 0.563), MR-Egger (OR = 0.162, 95% CI = 0.018–1.485, P = 0.206), and the WM method (OR = 0.955, 95% CI = 0.565–1.613, P = 0.862). Meanwhile, Cochran’s Q test did not indicate any noteworthy heterogeneity (P = 0.45). A leave-one-out analysis did not identify any single SNP significantly influencing our MR estimates (**Supplementary Figure 2B**). The MR-Egger intercept was 0.0019 (P = 0.054), indicating the absence of detectable horizontal pleiotropy (**Supplementary Table 3**). In the MR-PRESSO examination, no significant horizontal pleiotropy or outliers were detected (global test P = 0.097). It is worth noting that the power to detect the association between CeD and the IVs of ADHD was modest, at 15.00% (**Supplementary Table 4**). For a visual representation of our study data, please refer to the forest plots, leave-one-out plots, scatterplots, and funnel plots displayed in **Supplementary Figures 1**, **2**.

## Discussion

Our results clearly demonstrate that there is no significant link between CeD and an increased risk of developing ADHD. Moreover, the evidence supporting a causal connection between CeD risk and the occurrence of ADHD is scant. Nevertheless, it’s important to acknowledge that the precision of these findings might be somewhat diminished due to constraints in statistical power.

In 2015, a systematic review delved into the ongoing debate surrounding the potential link between CeD and ADHD. Intriguingly, its conclusions diverged from the consensus, finding no apparent correlation (47). But up until now, prior to this review, numerous observational studies had put forth compelling evidence demonstrating a substantial association between CeD and ADHD. A population-based study, after accounting for factors such as country of birth, parental education, birth weight, Apgar score, and psychiatric history, found that children with celiac disease had a 1.2-fold higher risk of ADHD compared to the general population (95% CI: 1.0–1.4) (48). In a substantial prospective cohort study, individuals with CeD had a higher ADHD risk compared to matched counterparts (HR = 1.19, 95% CI: 0.99–1.42). Stratified analyses by sex, age, calendar year, and follow-up time further confirmed this elevated risk (18). The findings from a meta-analysis lent further weight to these observations (16). On the other hand, in some other observational studies, the prevalence of celiac disease in populations with ADHD does not seem to be significantly different from that in control populations, and the findings do not support the view that CeD is more common in kids with ADHD than in kids without the disorder (49).

Our conclusion diverges from previous observational studies due to several critical factors: (i) The MR analysis approach carefully selects appropriate genetic variations (SNPs) as instrumental variables, leveraging GWAS databases to effectively mitigate the influence of confounding factors such as lifestyle and social environment on disease outcomes. CeD treatment solely involves a lifelong gluten-free diet (GFD) (50), but an imbalanced GFD can elevate the risk of obesity (51). An MR analysis has highlighted a positive causal link between obesity and a higher risk of ADHD (52). (ii) Since studies have demonstrated how DNA methylation or copy number variants can independently alter the risks of AD or ADHD, it is possible that the genetic relationship between the two could be mediated by epigenetic changes in copy number variations (53–56). (iii) Our study benefits from a large sample size in the MR analysis, enhancing its persuasiveness compared to earlier observational studies with smaller samples. Moreover, previous studies relied on cohort designs, which were relatively simplistic and lacked the ability to differentiate between causality and chronological order and were often constrained by geographical limitations. (iv) The disparities in our study findings compared to previous research linking CeD and ADHD may stem from differences in population inclusion and diagnostic criteria for the disease. Our study encompassed a broad spectrum of European countries, incorporating various European ethnic groups, whereas previous studies often focused on large cohorts within a single country or non-European populations (17, 18, 57). Furthermore, the GWAS database we used for our research followed strict guidelines for selecting case samples. For instance, in the case of CeD, diagnostic information encompassed not only clinical symptomatology and serological markers but also histopathological identification (24, 26). On the other hand, some past studies might have just used quick diagnostic tests for CeD in the absence of such thorough diagnostic protocols (57, 58).

Our research boasts several distinct advantages. Firstly, it represents the pioneering use of Mendelian randomization to investigate the relationship between CeD and ADHD. Our methodological contribution lies in systematically employing genetic instruments as proxies for modifiable risk factors, thereby enabling us to infer causality and mitigate common biases encountered in traditional observational studies. In addition, we conducted sensitivity tests to ensure the robustness of our final results. Secondly, we conducted bidirectional Mendelian randomization analyses, a valuable approach for examining potential reverse causal associations and enhancing the stability and reliability of our findings.

### Limitations of the study

However, this study has several limitations that should be taken into consideration. Firstly, addressing epigenetic factors such as DNA methylation, non-coding RNA regulation, and chromatin remodeling poses challenges in MR studies (43). Secondly, the phenomenon known as the winner’s curse could potentially inflate the genetic associations between exposure and outcome (59). To mitigate this bias, we employed four different MR methods to assess the MR estimates. Fortunately, these methods yielded consistent results, indicating that the winner’s curse only marginally affected our MR analysis. Thirdly, the statistical power of our primary analyses fell below the recommended threshold of 80%. This could be attributed to the limited variability in exposure explained by the chosen instrumental variables (37). Hence, caution should be exercised when interpreting the findings. Fourthly, the majority of individuals in the analyzed databases had European ancestry, limiting the generalizability of our findings to populations with more diverse ethnic backgrounds. Lastly, our ability to conduct additional subgroup analyses was constrained due to the unavailability of complete demographic and clinical data. Specifically, as ADHD is more prevalent in males, the lack of gender-stratified statistics in the database prevented us from assessing the impact of ADHD on the risk of CeD in different age groups and genders (60). This limitation may introduce bias into the study results.

## Conclusion

In conclusion, our MR investigation failed to establish a causal link between CeD and ADHD. Furthermore, we did not detect any compelling evidence supporting an association between these two conditions. To bolster future MR studies, we anticipate the availability of more extensive datasets, particularly those encompassing related factors, as the evidence in our study was hampered by its limited statistical power. To validate our findings in a practical context, additional mechanistic research, alongside large-scale, multicenter, and longitudinal studies, is imperative.

## Data availability statement

The datasets presented in this study can be found in online repositories. The names of the repository/repositories and accession number(s) can be found in the article/**Supplementary Material**.

## Ethics statement

Ethical approval was not required for the study involving humans in accordance with the local legislation and institutional requirements. Written informed consent to participate in this study was not required from the participants or the participants’ legal guardians/next of kin in accordance with the national legislation and the institutional requirements.

## Author contributions

JC: Conceptualization, Data curation, Methodology, Visualization, Writing – original draft. QZ: Data curation, Visualization, Writing – review & editing. LL: Methodology, Supervision, Writing – review & editing. ZX: Conceptualization, Funding acquisition, Project administration, Resources, Supervision, Writing – review & editing.
