# Supplementary material for: Celiac disease and attention-deficit/hyperactivity disorder: a bidirectional Mendelian randomization analysis
Source: Front Psychiatry. 2024 May 29;15:1291096. doi: 10.3389/fpsyt.2024.1291096 (PMC11167073; doi:10.3389/fpsyt.2024.1291096)
Supplement: Supplementary file 1 [file DataSheet_1.docx]

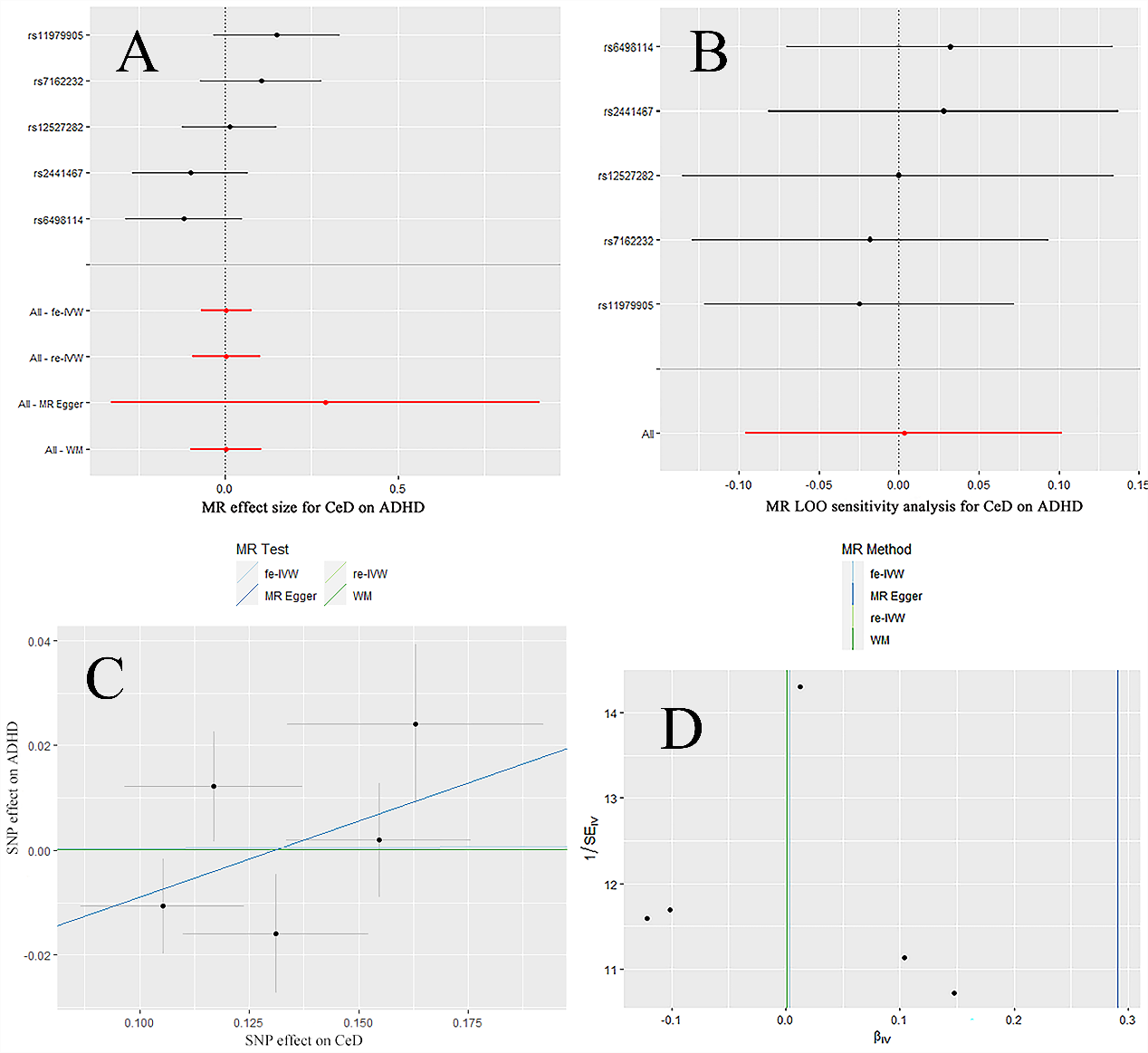


Supplementary Figure 1: Forest plot (A), leave-one-out analysis (B), scatter plot (C) and funnel plot (D) of the effect of CeD on ADHD. MR, Mendelian Randomization; SE, standard error; β, beta; IV, instrumental variables; fe-IVW, fixed effect inverse variance weighting; re-IVM, random effects inverse variance weighting; WM, weighted median; SNP, single nucleotide polymorphism; LOO, leave-one-out.


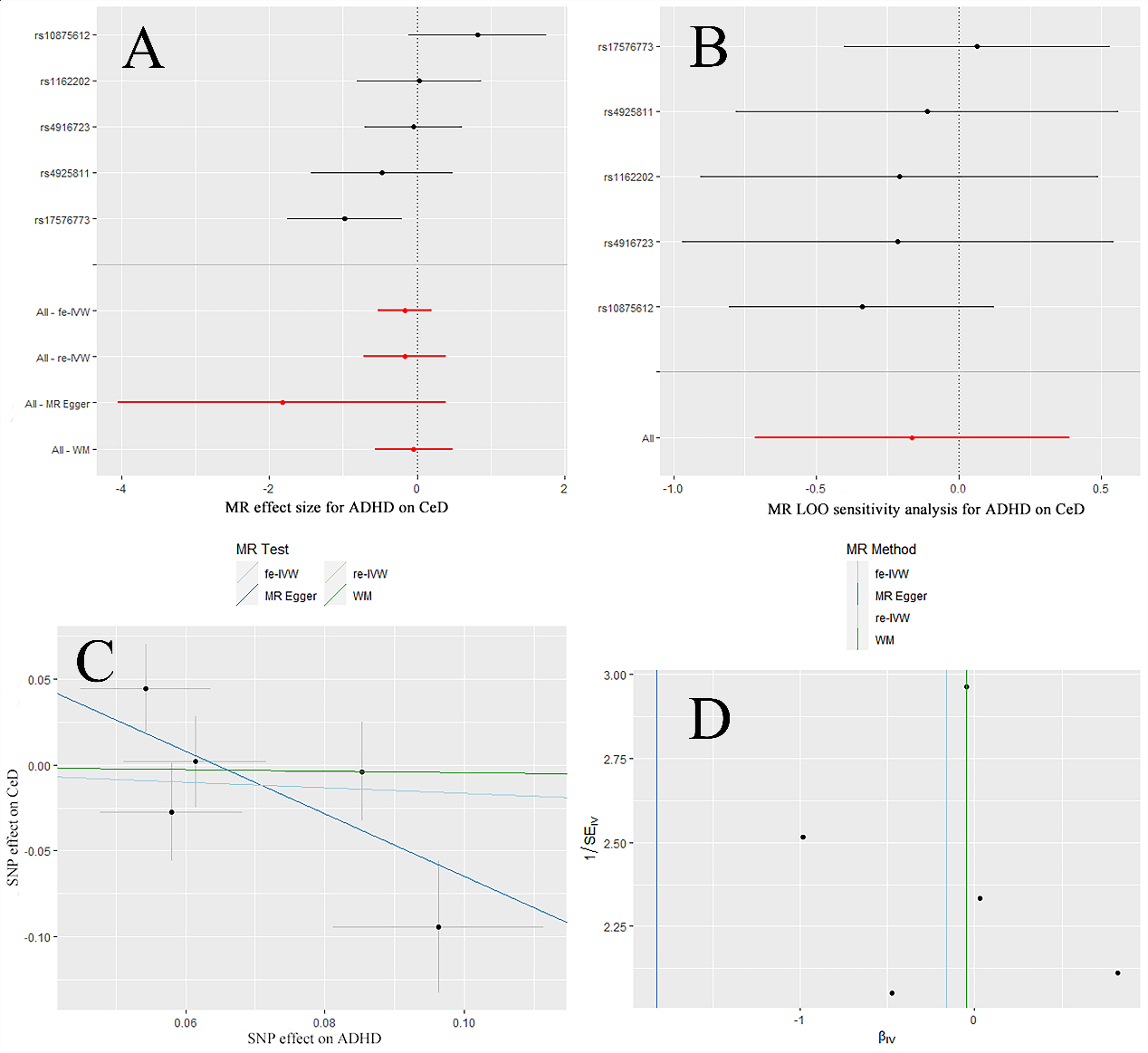


Supplementary Figure 2: Forest plot (A), leave-one-out analysis (B), scatter plot (C) and funnel plot (D) of the effect of ADHD on CeD. MR, Mendelian Randomization; SE, standard error; β, beta; IV, instrumental variables; fe-IVW, fixed effect inverse variance weighting; re-IVM, random effects inverse variance weighting; WM, weighted median; SNP, single nucleotide polymorphism; LOO, leave-one-out.
